# Small extracellular vesicles from human adipose-derived mesenchymal stromal cells: a potential promoter of fat graft survival

**DOI:** 10.1186/s13287-021-02319-4

**Published:** 2021-05-03

**Authors:** Aizhen Chen, Shijie Tang, Jiawei He, Xiangyu Li, Guohao Peng, Haoruo Zhang, Jinghua Chen, Liangwan Chen, Xiaosong Chen

**Affiliations:** 1 Department of Plastic Surgery, Fujian Medical University Union Hospital, Fuzhou, China; 2 Department of Plastic Surgery and Regenerative Medicine Institute, Fujian Medical University, Fuzhou, China; 3 Department of Stem Cell Research Institute, Fujian Medical University, Fuzhou, China; 4 Department of Pharmaceutical Analysis, the School of Pharmacy, Fujian Medical University, Fuzhou, China; 5 Department of Cardiac Surgery, Fujian Medical University Union Hospital, Fuzhou, China

**Keywords:** Small extracellular vesicles, Human adipose-derived mesenchymal stromal cells, Fat graft, Angiogenesis

## Abstract

**Background:**

Small extracellular vesicles (sEVs) with genetic information secreted by cells play a crucial role in the cellular microenvironment. In this study, our purpose is to explore the characteristics of the small extracellular vesicles of human adipose-derived mesenchymal stromal cells (hADMSC-sEVs) and studied the role of hADMSC-sEVs in improving the survival rate of grafted fat.

**Methods:**

In the present study, we used the transmission electron microscopy, nano-tracking analysis, nanoflow surface protein analysis, and zeta potential value to identify sEVs. SEVs’ trajectory was traced dynamically to verify whether hADMSC-sEVs can be internalized into human umbilical vein endothelial cells (HUVECs) in vitro at different times. The angiogenic property of hADMSC-sEVs was observed by measuring the volume, weight, and histological analysis of the grafted fats in nude mouse models.

**Results:**

Our research showed that the hADMSC-sEVs were sEVs with double-layer membrane structure and the diameter of which is within 30–150 nm. hADMSC-sEVs exert biological influence mainly through internalization into cells. Compared with the control group, the hADMSC-sEVs group had a significantly higher survival rate of grafted fat, morphological integrity, and a lower degree of inflammation and fibrosis. And immunohistochemistry showed that hADMSC-sEVs significantly increased the neovascularisation and the expression of CD34, VEGFR2, and Ki-67 in the graft tissue.

**Conclusions:**

As a potential nanomaterial, hADMSC-sEVs have been explored in the field of cell-free application of stem cell technology. hADMSC-sEVs promoted the survival of grafted fats by promoting the formation of new blood vessels, which is another promising progress in the field of regenerative medicine. We believe that hADMSC-sEVs will have a broad application prospect in the field of regenerative medicine in the future.

**Supplementary Information:**

The online version contains supplementary material available at 10.1186/s13287-021-02319-4.

## Background

Small extracellular vesicles (sEVs) are 30–150 nm membranous vesicles actively released by cells [1], containing various kinds of molecules—proteins, mRNA, miRNA, lipids—that are actively being studied as potential biomarkers and play essential roles in intercellular communication [2]. In particular, sEVs derived from adipose-derived mesenchymal stromal cells (hADMSC) have strong therapeutic potential and may offer a new therapeutic strategy [3]. Upon the literature review, we found that sEVs can promote angiogenesis [4, 5] and account for the beneficial paracrine effects of hADMSCs therapy [6]. Previous studies have confirmed that hADMSC-sEVs can effectively attenuate myocardial ischemia/reperfusion injury [7] and accelerate cutaneous wound healing [8].

In clinical, autologous fat grafting is playing an increasingly important role in the esthetic and reconstructive field for its extensive utility for soft tissue rejuvenation, volume augmentation, and body contouring. In contrast, the unpredictable fat resorption and low survival rates limit its further application and development [9, 10]. Mounting data have demonstrated that adequate neovascularization is vital for the survival and maintenance of grafted fat [11]. Therefore, many approaches have been developed to promote angiogenesis and improve fat graft retention. Among these, co-transplantation of autologous adipose tissue with hADMSCs or stromal vascular fraction (SVF), known as cell-assisted lipotransfer (CAL) technique [12], can enhance the survival rates by stimulating angiogenesis through a paracrine effect [13]. Adipose tissue seems to be the most advantageous tissue from which to isolate mesenchymal stromal cells because of its abundancy, subcutaneous location, and the need for less invasive techniques [14]. Furthermore, the primary dilemma of stem cell therapy lies in its difficultly to extend clinical applications for its safety concerns [15, 16]. Given that, we turned to explore a kind of biomaterial, which were equipped with the function of cells but no cells’ framework and maybe promising in clinical application. Based on the access to previous researches, we hypothesized that hADMSC-sEVs could be a kind of biomaterial applying in promoting vessel reconstruction after transplantation, with satisfactory biocompatibility and retention.

In this context, we extracted sEVs from the fourth passage of hADMSCs and identified the characteristics of hADMSCs and hADMSC-sEVs. Then, we explore the mechanism of hADMSC-sEVs’ exerting influence in this biological progress. We chose a nude mice fat grafting model to identify whether hADMSC-sEVs could potentially promote angiogenesis after fat grafting and studied the underlying mechanism of hADMSC-sEVs’ effect in improving the retention of fat graft.

## Materials and methods

### Animal maintenance

All animal protocols were implemented under the Animal Ethical Committee of Fujian Medical University’s supervision and approval. Eighteen male nude mice (6 weeks of age) were raised in the Experimental Animal Center of Fujian Medical University. Animals were kept in cages individually after grafting and maintained under ambient temperature.

### Cell culture

We obtained lipoaspirates from three healthy female patients who underwent liposuction of the thigh at the Department of Plastic Surgery, Fujian Medical University Union Hospital. The donors ranged in age between 25 and 45 years. All participants signed the informed consent. This study was approved by the Ethics Committee of Union hospital of Fujian Medicine University and performed following the principles described in the Declaration of Helsinki. hADMSCs were isolated as previously described [15]. Briefly, the lipoaspirate was washed with phosphate-buffered saline (PBS) and digested with 0.25% collagenase I (Sigma-Aldrich, St. Louis, MO, USA). After filtration and centrifugation, the cell pellet was resuspended in Dulbecco’s modified Eagle’s medium (DMEM) (Hyclone, USA) supplemented with 10% fetal bovine serum (FBS, Gibco, Carlsbad, CA, USA) and then cultured in an incubator with 37 °C and 5% CO_2_. The seeding density of cells was 5 × 10^5^/25 cm^2^. The fourth passage of hADMSCs, cultivated until around 80% confluency, was used in future experiments. hADMSCs from different donors were cultivated or kept separately. The collected hADMSCs were observed by inverted microscope (CNOPTEC, Chongqing, P. R. China) and characterized by osteogenic and adipogenic induction and flow cytometry (BD Biosciences, San Jose, CA, USA).

### Identification of hADMSCs

We chose the fourth-passage hADMSCs, which were in the state of logarithmic growth. When the cells grow to more than 80% confluence, we added adipogenesis induction media (a mixture of 1 μmol/L dexamethasone, 10 μmol/L insulin, 200 μmol/L indomethacin, 0.5 mmol/L isobutylmethylxanthine, and completely medium) and changed the medium every 3 days. Oil Red Assay kit (KeyGEN BioTECH, Jiangsu, China) was for lipid droplets staining according to the manufacturer’s specifications, and the results were observed under a microscope after 2 weeks.

Similarly, we used the fourth-passage hADMSCs which proliferated in logarithmically and added osteogenic induction media (a mixture of 10 mmol/L β-glycerol sodium phosphate, 0.1 μmol/L dexamethasone, 50 μmol/L vitamin C, and complete medium) after the cells grew to more than 80% confluence. Accordingly, we changed the medium every 3 days and used an alkaline phosphatase calcium cobalt staining kit (KeyGEN BioTECH, Jiangsu, China) for staining cells after 3 weeks according to the manufacturer’s instructions, and the results were observed under the microscope.

### Flow cytometry

The fourth passage of hADMSCs was selected for flow cytometry analysis for phenotypic identification of mesenchymal stromal cells. CD29, CD90, CD31, and CD45 along with related isotype controls (Abcam, Cambridge, UK) were used for hADMSCs’ immunofluorescence staining. Flow cytometry was performed by using the BD Accuri C6 System (BD Biosciences, San Jose, CA, USA).

### Acquisition of hADMSC-sEVs

hADMSC-sEVs were collected and purified according to the following processes. We selected the fourth-passage hADMSCs to extract sEVs. After cells’ confluency reaching 70–80%, hADMSCs’ culture medium was replaced with serum-free low glucose DMEM for 48 h to collect cells’ supernatant. To isolate and remove cell particles, dead cells, and cell debris of the obtained supernatant, we performed a series of differential centrifugal precipitation (300×*g* for 10 min, 2000×*g* for 10 min, and 10,000×*g* for 30 min). The supernatant removed the sediment was then filtered through a 0.22-μm filter (Millipore, USA) to remove the large extracellular vesicles further and ultracentrifuged at 100,000×*g* for 70 min by using the High-Speed Refrigerated Centrifuge (Beckman Coulter, USA). The supernatant was discarded, and the precipitation was resuspended with PBS. Finally, the suspension was ultracentrifuged at 100,000×*g* for 70 min again, and sEVs were obtained after precipitation collection. The obtained sEVs concentration was measured with bicinchoninic acid (BCA) protein detection kit (Beyotime, Shanghai, China) and stored at − 80 °C for further use. All centrifugations are operated at 4 °C.

### Nanoparticle tracking analysis

Particle size, particle size distribution, and concentration of sEVs were identified by nanoparticle tracking analysis (NTA) (NanoFCM, China). Compared with polystyrene beads (RI = 1.59), in the Nano-FCM system, monodisperse silica nanoparticles (RI = 1.46) are employed as the reference to calibrate the size of EVs. In the nanoFCM system, the detection efficiency is 100%. Particle concentration can be determined via single-particle enumeration, which defines the particle concentration of the number of particles collected in a given period. Finally, the size, distribution, and total concentration of EVs were calculated by NTA software.

### Zeta potential assay of sEVs

Zeta potentials of sEVs were measured three times using a Nano laser particle size analyzer (Litesizer 500, Anton Paar, Austria). Data were collected and analyzed using Anton Paar Kalliope software.

### Transmission electron microscopy

EVs were imaged by transmission electron microscopy (TEM) to verify their morphology. The sample with a volume of 5 μl (366 μg/ml) was prepared and dropped on the sealing film. Covered with a copper mesh and stood for 20 min so that the copper mesh fully absorbed sEVs. The copper mesh with sEVs adsorbed was transferred to 4% paraformaldehyde for fixation for 5 min. Then using 50 μl of 2% uranyl acetate to stain negatively with copper mesh for 5 min, and then copper mesh was dried at room temperature for 30 min. Finally, using FEI transmission electron microscopy (FEI Tecnai G2, USA) for imaging at 100 kV.

### Scanning electron microscopy

Take 20 μl sEVs samples and freeze-dry them in a freeze dryer for 16 h. Then, the lyophilized sEVs powder was evenly dispersed on the conductive tape of the sample holder, and the sample holder was placed in the gold evaporation chamber for ion sputtering gold plating. Finally, the shape and quantity of sEVs were observed under a high-low vacuum scanning electron microscope (SEM) (FEI QUANTA 450, USA), and the images were taken and recorded.

### Nanoflow analysis

Taking 30 μL of sEVs diluent and adding FITC Mouse Anti-Human CD9, CD81 (BD, Franklin Lake, New Jersey, USA) and FITC Mouse IgG (BioLegend, San Diego, USA) to mix, then incubating at 37 °C for 30 min in the dark, adding 1 ml of pre-cooled PBS, 110,000×*g* ultracentrifuging for 70 min. Carefully remove the supernatant, resuspend it in 50 μL of pre-cooled PBS, and detect the protein index results with a NanoFCM instrument (NanoFCM, China).

### Internalization of hADMSC-sEVs into human umbilical vein endothelial cells (HUVECs)

The hADMSC-sEVs were labeled with a PKH67 dye (Sigma Aldrich, USA) for 4 min. Then, Bovine Serum Albumin (BSA) was used to terminate staining. The excess dye after labeling was removed by ultracentrifugation at 100,000*g* for 1 h at 4°. HUVECs (Cellcook, Guangzhou, China) and hADMSC-sEVs labeled with PKH67 were incubated in 37 °C with serum-free medium for 6 h and 12 h. Meanwhile, HUVECs and PKH67 dye were incubated in 37 °C for 6 h and 12 h as control. After fixation with 4% paraformaldehyde (PFA) for 30 min and staining with 4, 6-diamino-2-phenylindoles (DAPI, CST, USA), the samples were observed under a fluorescence microscope (Nikon, Japan).

### Histological analysis and immunostaining

The grafted fat paraffin-embedded sections were stained with hematoxylin, eosin, and immunohistochemistry. The sections were primarily incubated with rabbit anti-human and mouse CD34 (Abcam, USA), VEGFR2 (Abcam, USA), and Ki-67 (Abcam, USA) separately, followed by incubation with horseradish peroxidase-conjugated secondary antibody. Finally, the staining color was developed using the DAB Detection Kit (Maixin, Fuzhou, China). Histologic parameters were examined under a light microscope. HE staining was observed in 5 low magnification field analysis methods for the assessment of fat graft integrity, as evidenced by the presence of intact and nucleated adipocytes and the presence of cysts and vacuoles. Each parameter was graded by two observers independently on a semiquantitative scale ranging from 0 to 5 (0 = absence; 1 = minimal presence; 2 = minimal to moderate presence; 3 = moderate presence; 4 = moderate to extensive presence; and 5 = extensive presence).

### Animal studies

Six-week-old, male nude mice were obtained from the Laboratory Animal Center of Fujian medicine University (Fuzhou, P. R. China). The experimental protocol was approved by the Animal Ethical Committee of Fujian Medical University (Permit Number: FJMU IACUC 2019-0131). The mice were randomly assigned to hADMSC-sEVs or control groups (six mice in each group). A mixture of 0.4 ml of fat particles and 0.1 ml (10^10^ particles/ml) hADMSC-sEVs solution (hADMSC-sEVs group) or 0.1 ml PBS (control group) was injected subcutaneously into the back of nude mice (the fat particles was from the other patient who had liposuction of thighs). The mice were sacrificed at 1, 2, and 3 months after fat grafting. Grafted fat samples were harvested and measured by the weight, volume, hematoxylin-eosin (HE), and immunochemistry staining. The weights of fat grafts were determined by a balance and the volumes were measured by the liquid overflow method. The analysis of HE and IHC staning has been discussed above.

### Statistical analysis

Statistical analysis was performed using GraphPad Prism 8.0.1 software (GraphPad Software, Inc., La Jolla, CA, USA). Results were presented as the mean ± standard deviation (SD). The two-tailed Student’s *t* test was used to evaluate differences between groups. *P* < 0.05 was considered as statistically significant.

## Results

### Characterizations of hADMSCs

**Fig. 1:**
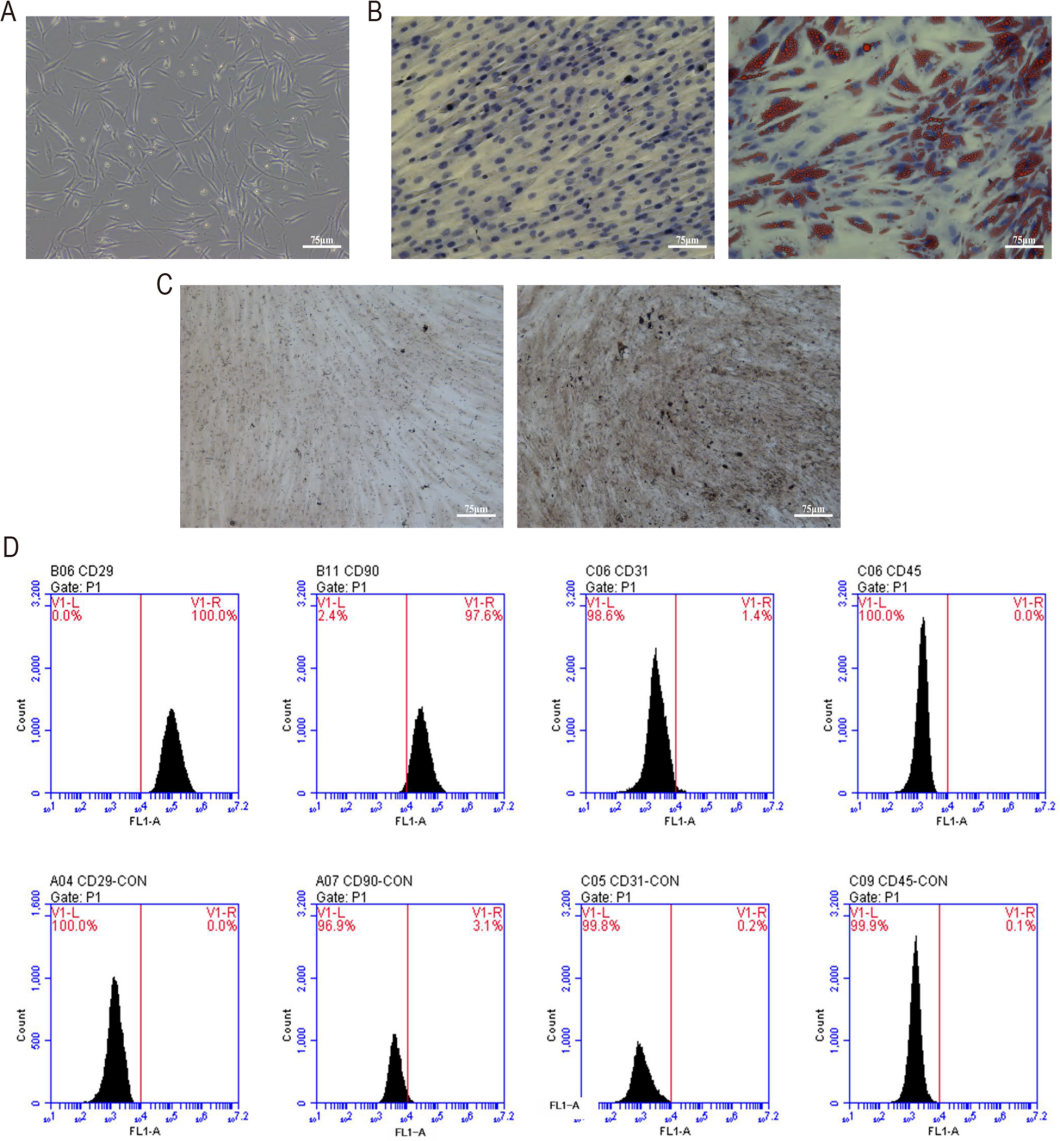
Characterizations of human adipose-derived mesenchymal stromal cells (hADMSCs). **a** Morphological features of the fourth-passage hADMSCs under light microscopy (× 100 magnification, scale bar =75 μm). **b** Representative images of Oil Red O staining (left: non-induced control group; right: induced group; × 200 magnification, scale bar = 75 μm). **c** Representative images of the alkaline phosphatase calcium cobalt staining (left: non-induced control group; right: induced group; × 200 magnification, scale bar =75 μm). **d** Flow cytometry analysis of cell surface marker CD29, CD90, CD31, and CD45 was showed in hADMSCs

Under light microscope, a characteristic morphology of slender spindle-like cells of fourth-passage hADMSCs was observed (Fig. 1a). Mature adipocytes and mineralized nodules can be formed from fourth-passage hADMSCs induced by adipogenic and osteoblastic induction media, respectively (Fig. 1b, right; c, right). The results of oil red O staining showed that there were red fat droplets with different sizes in the cells. And the alkaline phosphatase calcium cobalt method staining showed positive osteoblastic nodules. To determine the mesenchymal phenotype of hADMSCs, we investigated the purified hADMSCs by using flow cytometry analysis. Approximately 98% of hADMSCs were positive for CD29 and CD90, but negative for CD31 and CD45 (Fig. 1d). These suggested that the cultured cells were adipose-derived mesenchymal stromal cells [17].

### Characterizations of hADMSC-sEVs

**Fig. 2:**
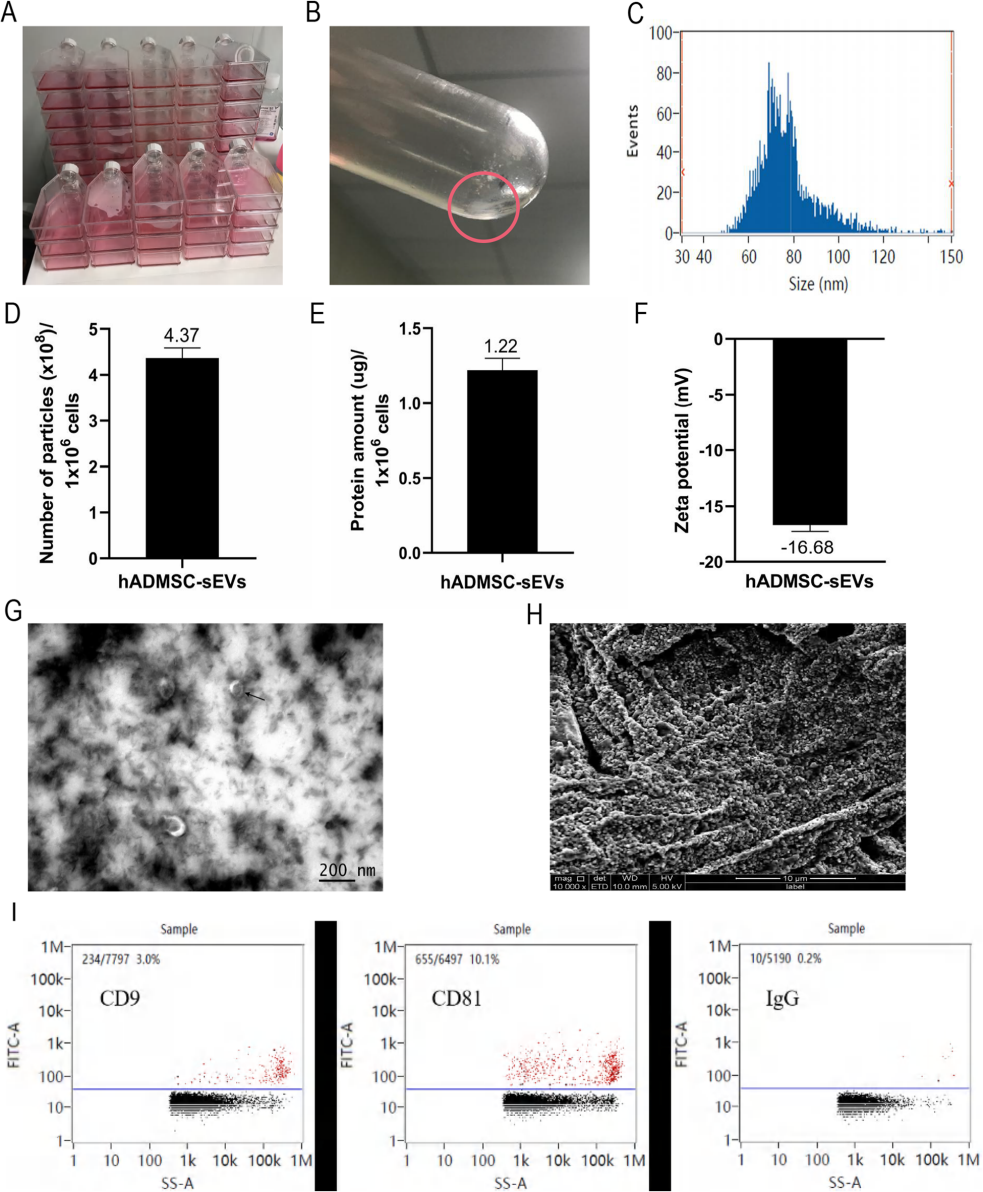
Characterizations of hADMSC-sEVs. **a** Gross observation: the number of T75 flasks for culturing cells at one time. **b** The gross visible sEVs precipitate was obtained from **a**. **c** NTA measured the size distribution of sEVs. **d** The ratio of the number of sEVs particles(× 10^8^) to 1 × 10^6^ cells. **e** The ratio of protein amount (μg) to 1 × 10^6^ cells. **f** The potential of hADMSC-sEVs was measured by Zeta potential. **g** Representative transmission electron microscopy image of ADMSC-sEVs with double circular vesicle structures was shown by directional arrow (× 100,000 magnification, scale bar = 200 nm). **h** Morphological characterization of sEVs with three-dimensional shapes under scanning electron microscopy (× 10,000magnification; scale bar = 10 μm). **i** Nanoflow analysis of the hADMSC-sEVs’ surface protein marker (CD9 and CD81), and IgG as positive controls

The classic ultracentrifugation method was used to obtain hADMSC-sEVs. We obtained the sEVs pellet of Fig. 2b from the cell supernatant of Fig. 2a after a series of differential centrifugation. According to the results of NTA data analysis, most of the sEVs’particle size ranged between 50 and 120 nm (Fig. 2c). We estimated the ratio of the number of sEVs particles (× 10^8^) to 1 × 10^6^ cells and the ratio of protein amount (μg) to 1 × 10^6^ cells through the measurement of NTA and previous BCA (Fig. 2d, e). It can be seen from the ratio of Fig. 2d and e that a large amount of cell-derived supernatant was needed to extract a sufficient amount of sEVs precipitate. Zeta potential measurement of sEVs showed that its average potential was − 16.68 mV (Fig. 2f). The morphology of sEVs was observed under transmission electron microscopy, which was a saucer-like membrane structure with a diameter of 50 ~ 120 nm (Fig. 2g). Under the scanning electron microscopy, the sEVs can be seen in a more objective and accurate spherical three-dimensional shape, with particle size similar to that of the transmission electron microscope (Fig. 2h). Specific markers CD9 and CD81 of EVs were confirmed to be expressed in hADMSC-sEVs [18], and IgG was used as a negative control (Fig. 2i).

### hADMSC-sEVs can be transferred to HUVECs

**Fig. 3:**
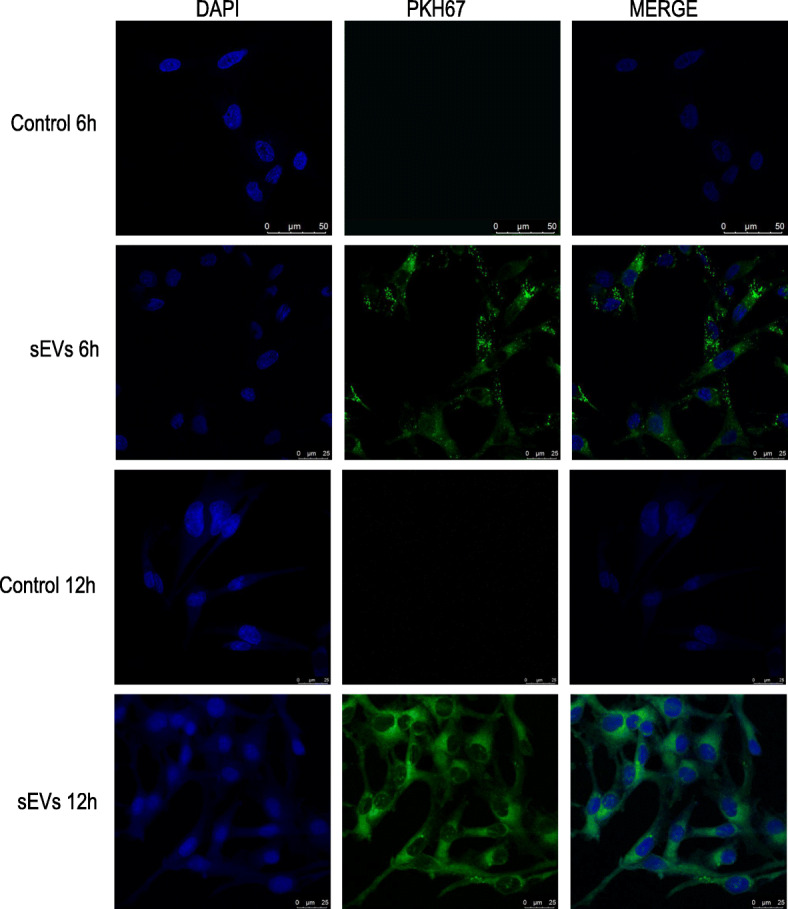
hADMSC-sEVs internalization to HUVECs. hADMSC-sEVs (labeled with PKH67 dye, green) and HUVECs (nuclei stained with DAPI) were co-incubated for 6 h and 12 h, respectively. In the control group, PKH67 dye was co-incubated with HUVECs for 6 h and 12 h, respectively. Representative fluorescence images were shown above(× 1000 magnification; scale bar = 25 μm)

As shown in Fig. 3, the PKH67-labeled hADMSC-sEVs and PKH67 dye were co-incubated with HUVECs for 6 h and 12 h, seperately. We can find that the PKH67-labeled hADMSC-sEVs were increasingly scattered around the nucleus of HUVECs as time went on. However, the control group of pure PKH67 dye did not find any green fluorescence accumulation. That was, only the substances with biological activity can enter the cells and the pure dye cannot through the cell membrane. The large stained bodies that we saw were the aggregation of a large number of sEVs labeled with pKH67 fluorescence, rather than individual tiny sEV, which means hADMSC-sEVs play their role mainly through internalization into cells. This result was mutually corroborated with related articles [19–21].

### hADMSC-sEVs improved the fat graft survival rate in the nude mouse model

**Fig. 4:**
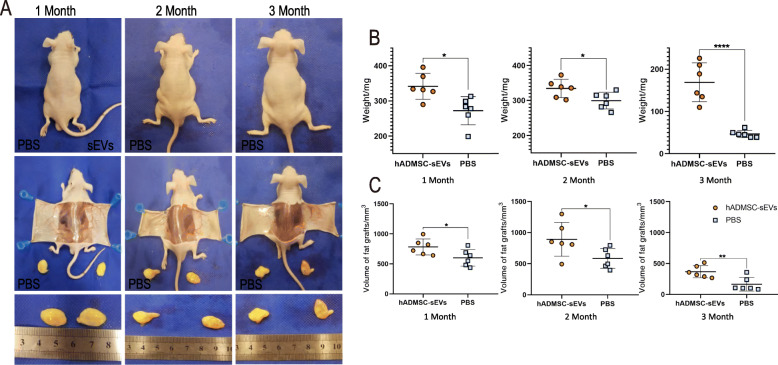
hADMSC-sEVs improved the fat graft survival rate in the nude mouse model. **a** Gross observation of the grafted fat demonstrated that the hADMSC-sEVs group (the left side of each picture) had larger graft sizes and heavier weight compared to that in the control group (the right side of each picture). **b**, **c** 1, 2, and 3 months after fat grafting, the weight (measured by the balance) and volume (measured by the liqiud outflow method) of grafted fat were significantly better in the hADMSC-sEVs group compared to the control group (*p < 0.05, **p < 0.01, ***p < 0.001,****p < 0.0001)

**Fig. 5:**
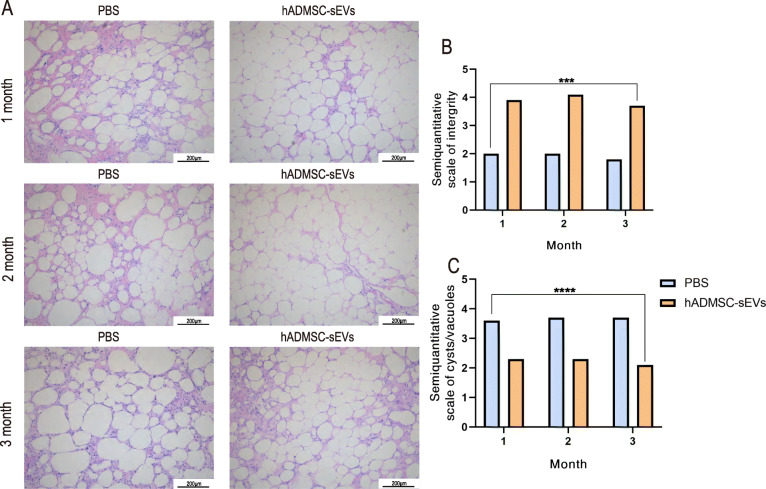
Histological changes in adipose tissue over time. **a** HE staining revealed that the grafted fat in the hADMSC-sEVs groups exhibited better survival and morphologic integrity (evidenced by the presence of intact and nucleated adipocytes and the presence of cysts and vacuoles) compared to the control group. **b**, **c** Semiquantitative scale of integrity and cysts/vacuoles of HE staining (***p < 0.001,****p < 0.0001)

To assess the beneficial effects of hADMSC-sEVs, we adopted a nude mouse model of fat grafting. The grafts were harvested 1, 2, and 3 months after fat grafting. Gross observation of the graft specimens demonstrated that the hADMSC-sEVs group had larger graft sizes compared to that in the control group (Fig. 4a). As shown in Fig. 4b, c, a significantly higher graft survival rate was observed in the hADMSC-sEVs group when compared with the control group from 1 to 3 months after fat grafting (*P* < 0.05), which was confirmed by the better quantitative results of weight and volume of grafted fat were better in the hADMSC-sEVs group compared to the control group, indicating a protective effect of hADMSC-sEVs on grafted fat survival. For micro-evaluation, we analyzed the HE staining, which revealed that the grafted fat in the hADMSC-sEVs groups exhibited better survival and morphologic integrity compared to the control group, as shown in Fig. 5a. We observed extensive cystic changes and fibrous septa in the control group. There were significant differences between the hADMSC-sEVs groups and control group in the histological evaluation of integrity, cysts/vacuoles, fibrosis, and inflammation (*P* < 0.05) (Fig. 5b, c).

### hADMSC-sEVs promoted neovascularization in the nude mouse model of fat grafting

**Fig. 6:**
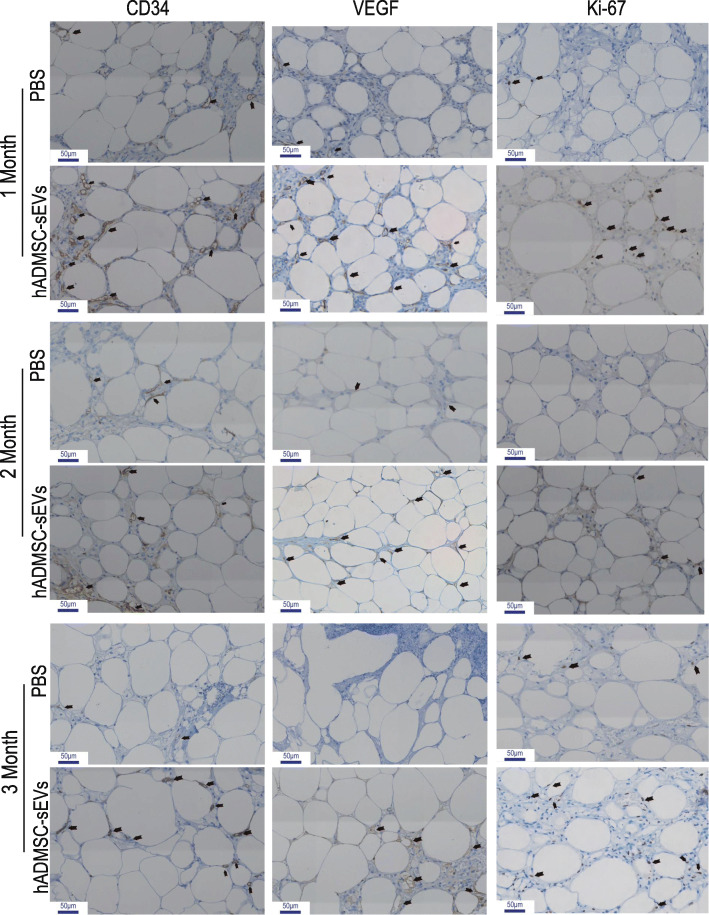
hADMSC-sEVs promoted neovascularization in the nude mice fat grafting model (rows means different staining and lines represents different groups and months). CD34 was selectively expressed in the small vessel endothelium membrane. The VEGF-kinase ligand/receptor signaling system plays a key role in vascular development and regulation of vascular permeability. The VEGFR2 was mainly localized in membrane. Signs of proliferation (nuclei stained by Ki-67) in the vessel endothelium (blue line at the bottom left of each figure represents scale bar; scale bar = 50 μm)

We used immunohistologic staining for histological evaluation. Studies have shown that vascularisation is crucial for fat survival and repassage [22]. So we measured capillary density within the grafts via immunohistochemical (IHC) staining of anti-CD34 antibody in tissue sections to evaluate the effect of hADMSC-sEVs on neoangiogenesis of grafted fat. Consistent with the HE staining and histologic examination, IHC staining showed a significant increase in CD34-positive (a specific marker of capillary [23]) in the hADMSC-sEVs group with the control groups (Fig. 6; See supplemental materials for the enlarged subsections of the images), indicating increased capillary density in the hADMSC-sEVs group. Together, these results showed that hADMSC-sEVs could effectively improve the vascularisation of the grafted fat.

### Mechanism of hADMSC-sEVs-mediated angiogenesis in vivo

The previous study has revealed that VEGF secreted by stromal cells stimulates the proliferation and survival of endothelial cells leading to the formation of new blood vessels [7]. We further evaluated the potential mechanism of hADMSC-sEVs in promoting survival of grafted fat by IHC analysis of anti-VEGFR2. The results demonstrated that the expression of VEGFR2 was increased in the hADMSC-sEVs group compared to the control group in 1, 2 and 3 months after transplantation (Fig. 6), suggesting that VEGF/VEGFR signaling pathway was involved in hADMSC-sEVs-mediated angiogenesis. We also semi-quantified endothelium cell proliferation by analyzing Ki-67 staining [24], and we found increased immunostaining of Ki-67 in the hADMSC-sEVs group compared with the control group (Fig. 6), suggesting that the proliferation of vascular endothelial cells were enhanced after hADMSC-sEVs treatment.

## Discussion

The study of EVs spans decades. In recent years, it has been brought to the fore, which is mainly due to the remarkable advancement of the identification of EVs, and related mechanisms have been continuously explored [18]. In particular, hADMSC-sEVs have shown great potential in a variety of disease models [25–28].

In this study, we used a classic ultracentrifugation method to separate the target sEVs, which obtained sEVs with higher purity. After acquiring sEVs, we further analyzed the morphology, surface markers, and physical properties of hADMSC-sEVs in combination with various methods as follows. The results of two-dimensional and three-dimensional images showed that the sEVs we obtained were spherical double-layer membrane structures with a particle size range of about 50–150 nm, which was similar to other related literature [29]. Most of those vesicles (98%) were 50–120 nm based on NTA analysis, and the mean size was 77 nm, which was consistent with the acknowledged size range (30–150 nm) [30]. The above showed that the classic ultracentrifugation method could be used to enrich nano-level EVs at a higher purity. Among membrane proteins, we used CD9 and CD81 as positive markers and IgG as the negative control. CD9 and CD81 are usually associated with EVs and are often regarded as surface protein markers for EVs [31]. Through nanoflow analysis, we successfully detected the sEVs marker proteins CD9 (3.0%), CD81 (10.1%), and IgG (0.2%). In terms of physical properties, hADMSC-sEVs displayed negative zeta potential values. All these data indicated that the extracellular vesicles of hADMSCs we extracted are small EVs, which is the operational term referred to EVs’ size, actually [24].

In our animal model of fat grafting, we found an interesting phenomenon that the weight of the grafts was much less at 3 months of implantation both in the control and the hADSC-sVEs group compared to the other months studied. That can be explained according to the rationale of the progression of grafted fat. Which mainly includes cysts, nodules, calcification, necrosis, fibrosis, and survival, and the progress basically takes about 3 months to reach stability [15]. The reason why the weight did not drop significantly at 2 months was mainly because part of the fat necrosis formed cysts or nodules, which had not yet or had just begun to be absorbed. By 3 months, these inflammatory responses, calcification absorption, etc., tend to stabilize. The surviving fat body weight and volume also tend to stabilize. So we found that there was a significant decrease in body weight in the 3 months. We generally think that the body weight after the third month can represent the live fat cells that survived the grafted fat.

The relationship between sEVs and vascular repassage is the focus of research in the field of regenerative medicine. Studies have shown that sEVs can change the angiogenesis steps including proliferation, migration, and endothelial cell structure, as well as increase the expression of angiogenesis-related genes and the secretion of related proteins, including VEGFA, CXCL8, IL-6, FGF2, and miRNA-23a [32–35]. To test whether sEVs can improve the survival of grafted fat by promoting angiogenesis, we adopted the nude mice fat grafting model. Consistent with previous studies [32, 36], we demonstrated that fat grafts in the hADMSC-sEVs groups exhibited better survival and morphologic integrity compared to the control group.

Several factors are contributing to fat graft survival [9]. Since the grafted fat show lower tolerance for ischemia caused by devascularisation, it is quickly absorbed and replaced by fibrous tissues and oil sacs [37, 38]. The development of a neovascular supply, or angiogenesis, serves crucial homeostatic roles since blood vessels carry nutrients to tissues and organs and remove catabolic products [39]. Therefore, timely and adequate neoangiogenesis is essential for the survival of grafted fat [40, 41]. Substantial evidence demonstrates CD34 is expressed not only by MSC but by a multitude of other nonhematopoietic cell types, including vascular endothelial progenitors [42]. Therefore, we adopted CD34 as a marker of neovascularisation analysis. Our results showed a significant increase in CD34-positive rate in the hADMSC-sEVs group with the control groups (Fig. 6), indicating increased capillary density in the hADMSC-sEVs group. We can observe the improvement of angiogenesis in the hADMSC-sEVs group, and then we began to focus on the underlying mechanism of those improvements. Studies have revealed that VEGF is identified as a principal pro-angiogenic factor that enhances the production of new blood vessels from the existing vascular network [43]. While research suggested that VEGF undergoes alternative exon splicing that leads to multiple isoforms, which has different functions and affinities to the same targets [44]. So there is some concern of low positive rate or false negative rate of anti-VEGFA. That is the reason why we chose VEGFR but not secreted VEGF, because we think the up-regulation of receptors may reflect the real condition about enhancements. In addition, it is well established that VEGF/VEGFR2 signaling pathway plays a vital role in regulating the process of neoangiogenesis. And it was demonstrated that the lower-affinity, highly homologous VEGFR2 was the primary signaling receptor for VEGF [45]. We think the VEGF family has complicated and extensive participation on the VEGF signaling pathway. So we prefer to study VEGFR2. Theoretically, an increase in ligands can lead to upregulation of receptors. Therefore, if the expression of VEGFR2 increases, on the one hand, the upstream stimulus signal is enhanced, and on the other hand, the secreted VEGF (A, C, or D) is also in a state of high expression [39]. So we chose VEGFR2 as our research target. Our IHC analysis demonstrated elevated VEGFR2 expression in grafted tissue of the hADMSC-sEVs group. Therefore, the high expression of VEGFR2 in the hADMSC-sEVs group may account for its pro-angiogenic effects. For further verification and exploration on the mechanism about that if there were the circumstance on proliferation of endothelia, so we analyzed the Ki-67 IHC staining and found increased immunostaining of Ki-67 in the hADMSC-sEVs group compared with the control group, suggesting that hADMSC-sEVs treatment could enhance the proliferation of vascular endothelial cells.

As a potential type of nanomaterial, sEVs have attracted growing attention from researchers in different fields. Interdisciplinary integration and the use of nanotechnology continuously promote the development of sEVs. However, in the process of extraction, identification, and subsequent mechanism research of sEVs, we found that a large amount of supernatant was needed to extract only a little bit of sEVs (Fig. 2a and b). Therefore, how to obtain a large number of sEVs has become a critical preclinical and clinical research direction. Only by breaking the bottleneck of sEVs production can the clinical transformation and application of sEVs have infinite development possibilities. We think that there are two main ways to increase the output of sEVs. One is to increase the number of sEVs secreted by cells from the source, which is, microcarrier-based three-dimensional (3D) cell culture technology. The other is to reduce the loss of sEVs, which means in the process of extracting EVs, it is necessary not only to ensure its purity but also to minimize the loss of sEVs.

3D cell culture technology is a common strategy for large-scale adherent cell culture, which adopts a kind of device equipped with three-dimensional culture system based on a hollow fiber bioreactor, and a large amount of conditioned medium (CM) can be obtained by using this device. Studies have shown that compared with the traditional 2D culture, the total amount of sEVs in the 3D culture system has increased 19.4 times [46, 47]. Moreover, compared with 2D-sEVs, 3D-sEVs have no significant differences in surface markers, size, and shape. In particular, 3D-sEVs can significantly improve the symptoms of related diseases in animal models and are more effective than 2D-sEVs [48]. In conclusion, sEVs obtained by 3D cell culture are more in line with the needs of the body’s biological functions, which is an important measure for the clinical development of sEVs.

In terms of sEVs extraction, the mainstream sEVs extraction methods mainly include ultracentrifugation (UC), density gradient centrifugation (DGC), exclusion chromatography (SEC), ultrafiltration (UF), Immune capture (IC) and polymer precipitation (Precip) [49]. Among them, the most classic method is UC [50], but its main limitations are time-consuming and low yield. So people began the exploration of how to increase the output of sEVs further and reduce the cost while ensuring its purity. In recent years, preclinical studies have found that the extraction method of UF combined with SEC is superior to the ultracentrifugation method under the comprehensive conditions of purity, efficiency and cost [51–54]. In addition, in industrial production, mass production of high-quality sEVs is the most critical factor in its therapeutic applications. Among various separation methods, tangential flow filtration (TFF) is considered as an ideal method for industrial-scale production of sEVs [55–57]. TFF can provide GMP level sEVs from a large amount of CM [58]. Some studies have even shown that sEVs separated by TFF have higher yield and activity than those separated by ultracentrifugation [45, 59]. And the analysis of multiple batches of isolated MSC-sEVs showed that the TFF method could generate stable sEVs in a large volume of media. Therefore, TFF is suitable for large-scale production of high-quality sEVs that meet GMP requirements [48].

The present study had several limitations. First, the downstream molecules in the VEGF/VEGFR2 signaling pathway remain to be defined in our further investigation. Second, the theory of graft retention or endogenous adipose repassage is still being defined. Consequently, further studies are warranted to address this issue.

## Conclusion

Small extracellular vesicles, as a novel kind of nanoparticle without nuclear structure, do not show apparent side effects, such as immunogenicity or tumorigenicity when applied in animal models. Studies have found that EVs can replicate the function of the cells which they are derived. Our research has proved that hADMSC-sEVs play a considerable role in fat grafting nude mouse model. hADMSC-sEVs can promote neovascularization and increase the retention of grafted fat, whose mechanism may be explained by VEGF/VEGFR2 signal transduction. These findings indicate that hADMSC-sEVs can be regarded as a potential treatment option for fat transplantation. As a new type of nanomaterial, we need further and more in-depth studies to promote hADMSC-sEVs to apply in a broader range of diseases.

## Supplementary Information

**Additional file 1.** Supplemntal material (enlarged figures) hADMSC-sEVs promoted neovascularization in the nude mice fat grafting model (rows meaned different staining and lines represents different groups and months).

## Data Availability

The data that support the findings of this study are available from the corresponding author upon reasonable request.

## Abbreviations

ADMSCs: Adipose-derived mesenchymal stromal cells
DMEM: Dulbecco’s modified Eagle’s medium
PBS: Phosphate-buffered saline
sEVs: Small extracellular vesicles
hADMSC-sEVs: The small extracellular vesicles of human adipose-derived mesenchymal stromal cells
HUVECs: Human umbilical vein endothelial cells
MSCs: Mesenchymal stem/stromal cells
SVF: Stromal vascular fraction
CAL: Cell-assisted lipotransfer
FBS: Fetal bovine serum
BCA: Bicinchoninic acid
TEM: Transmission electron microscopy
SEM: Scanning electron microscope
NTA: Nanoparticle tracking analysis
BSA: Bovine serum albumin
PFA: Oaraformaldehyde
DAPI: 4, 6-diamino-2-phenylindoles
HE: Hematoxylin-eosin
SD: Standard deviation
CM: Conditioned medium
UC: Ultracentrifugation
DGC: Density gradient centrifugation
SEC: Exclusion chromatography
UF: Ultrafiltration
IC: Immune capture
Precip: Polymer precipitation
TFF: Tangential flow filtration
