# Supplementary figures and images for: Small extracellular vesicles from human adipose-derived mesenchymal stromal cells: a potential promoter of fat graft survival
Source: Stem Cell Res Ther. 2021 May 3;12:263. doi: 10.1186/s13287-021-02319-4 (PMC8091529; doi:10.1186/s13287-021-02319-4)

1 Month

PBS

hADMSC-sEVs

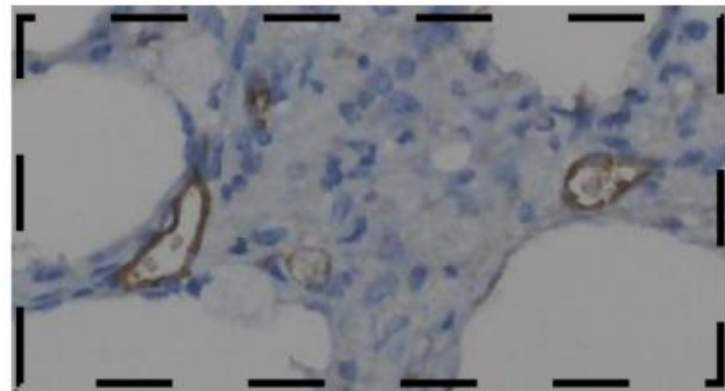

CD34

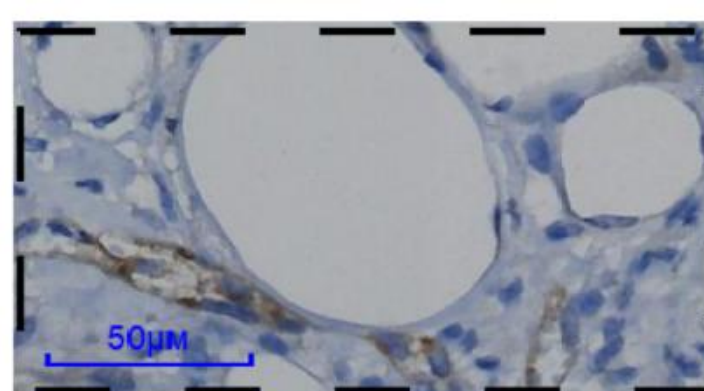

VEGF

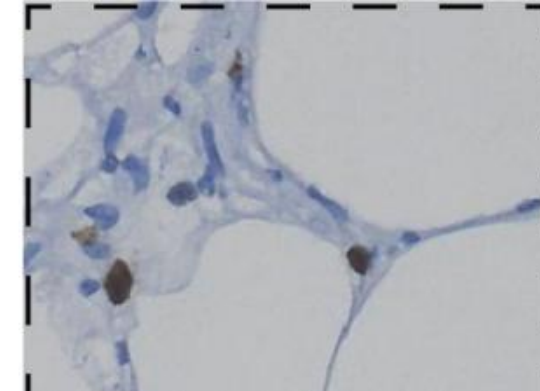

Ki-67

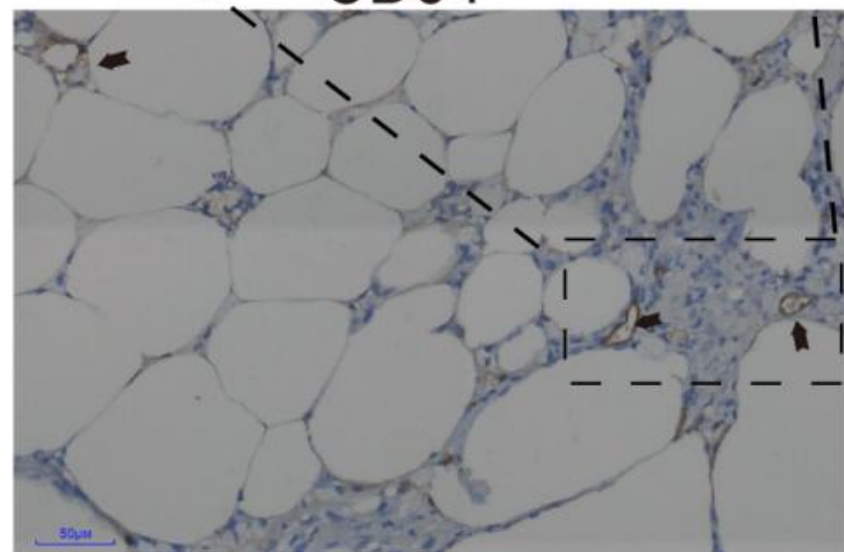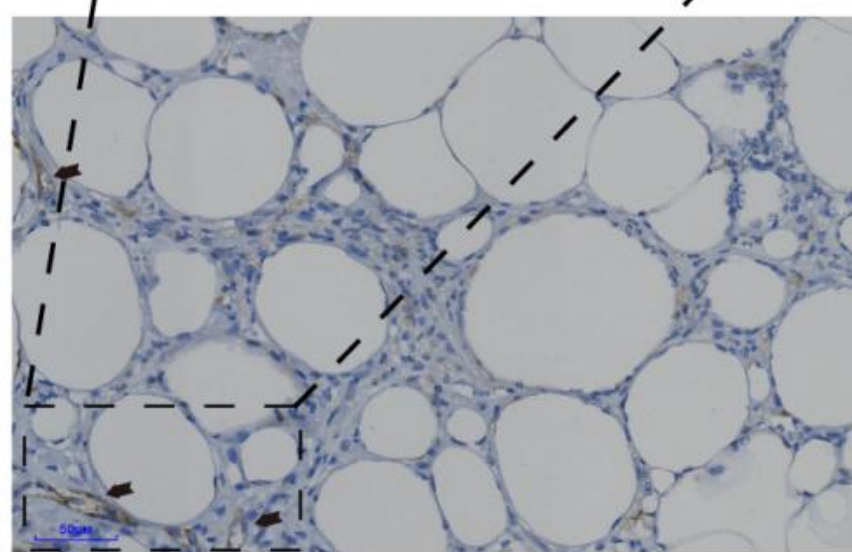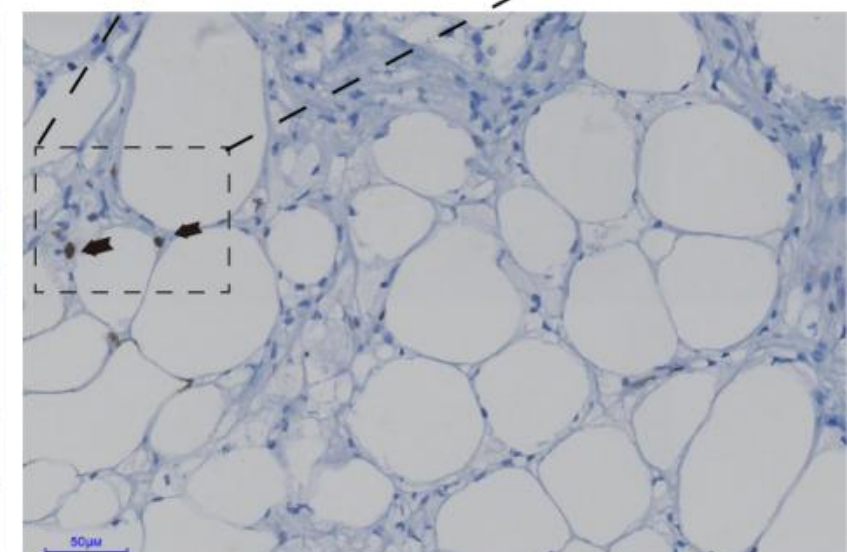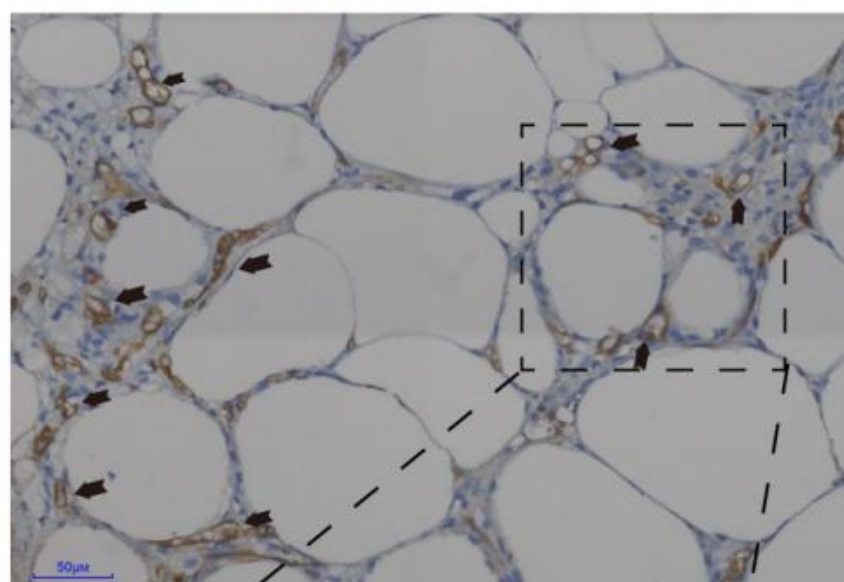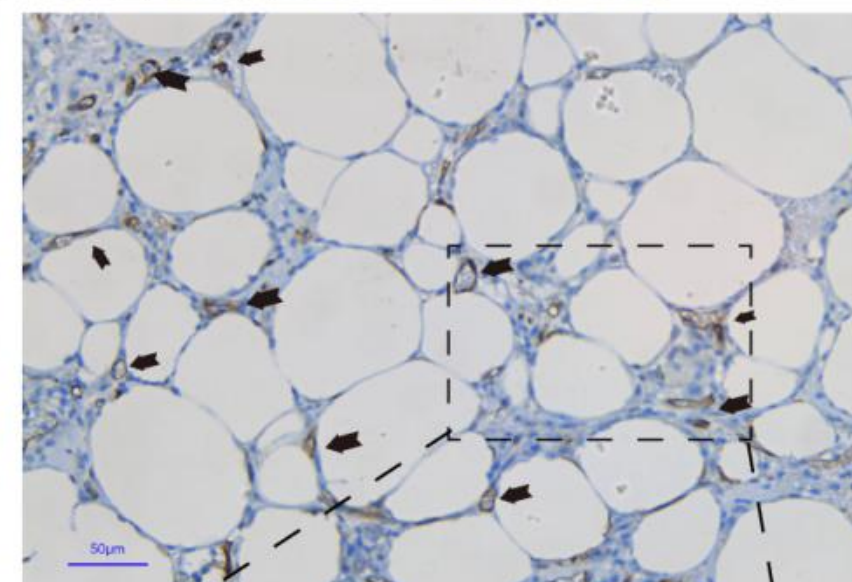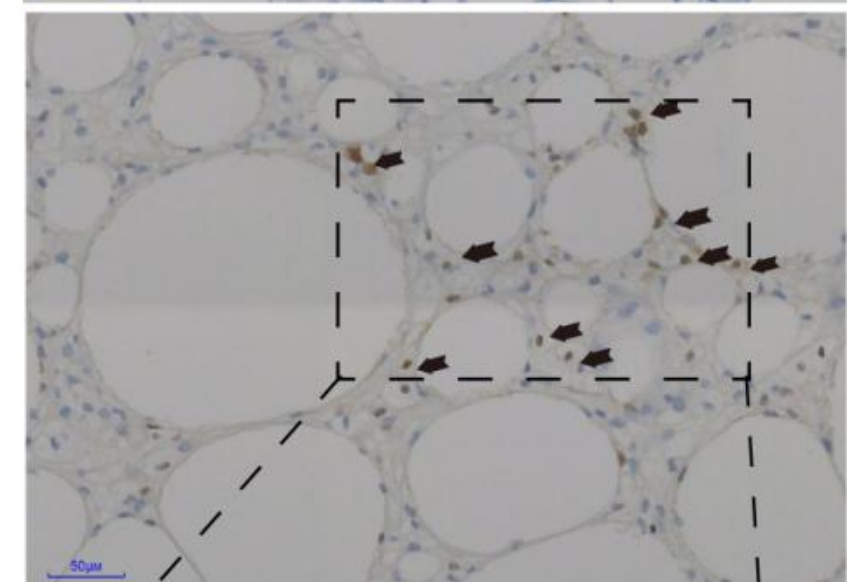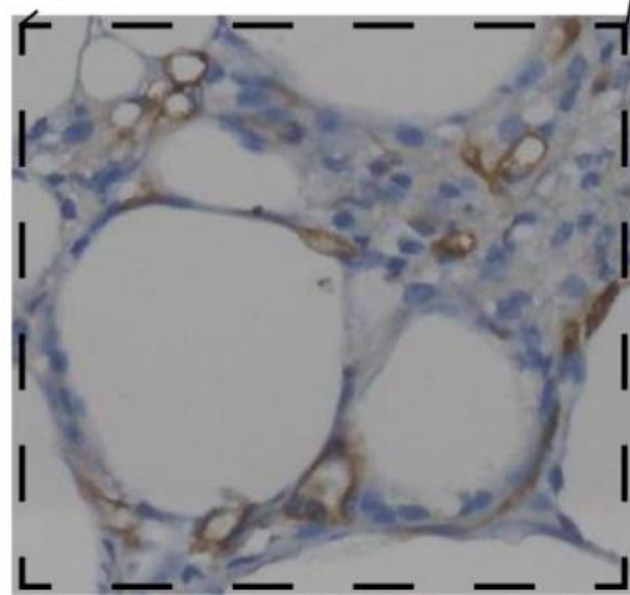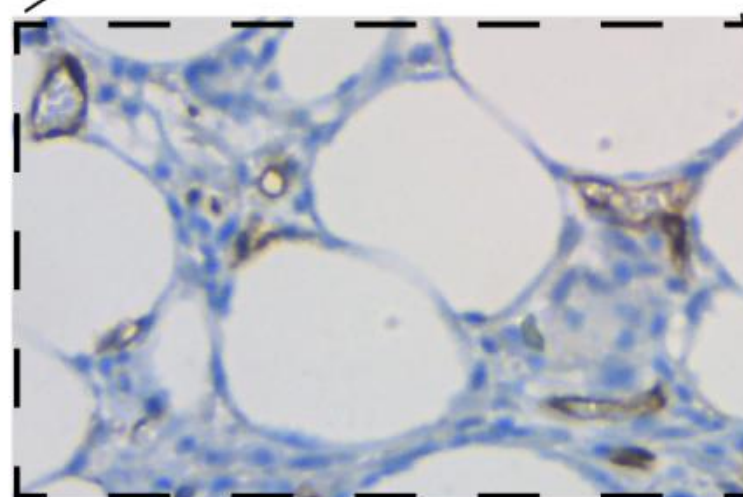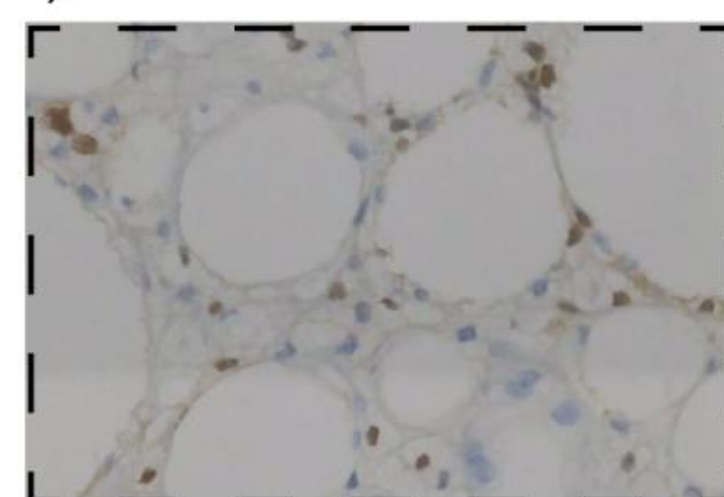

2 Month

PBS

hADMSC-sEVs

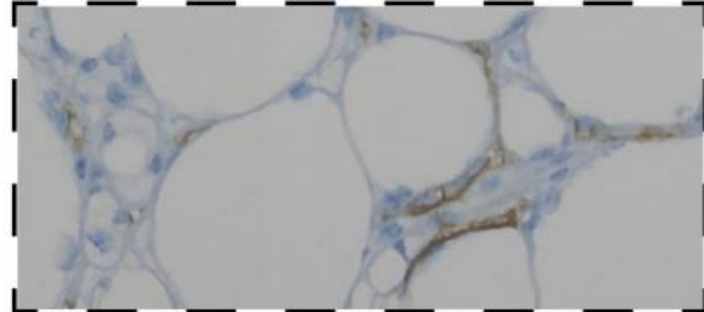

CD34

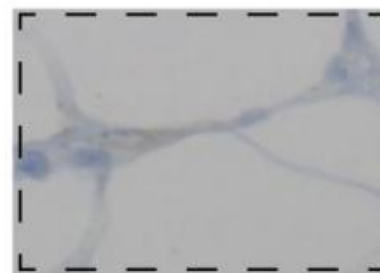

VEGF

Ki-67

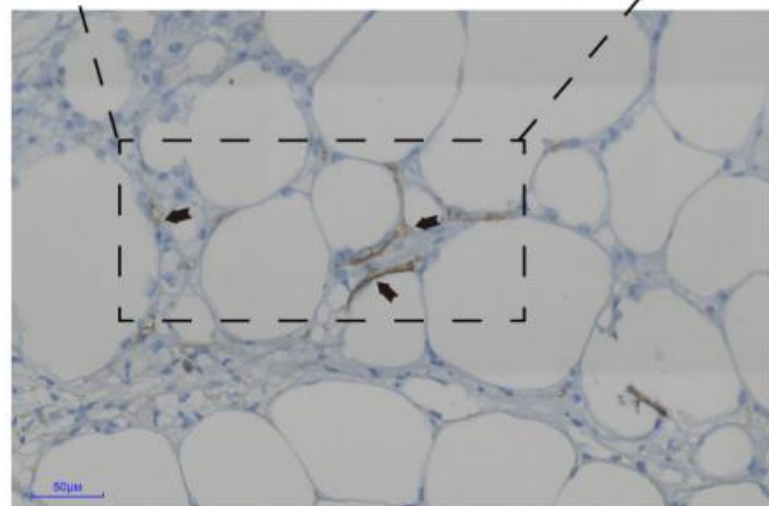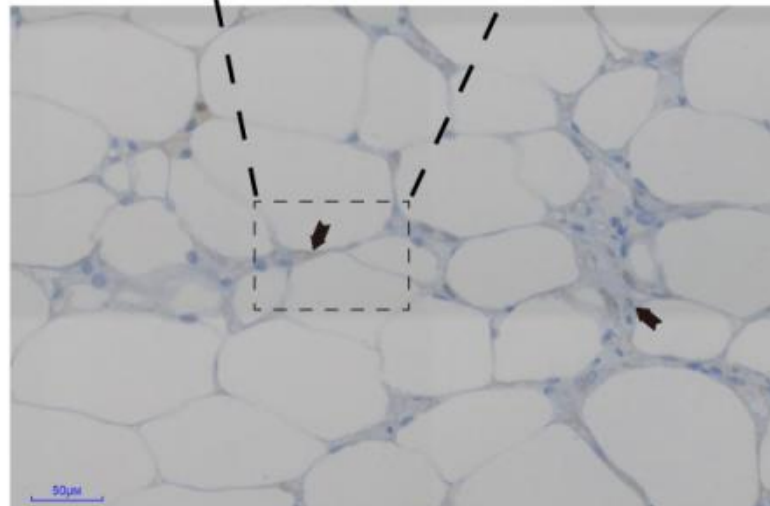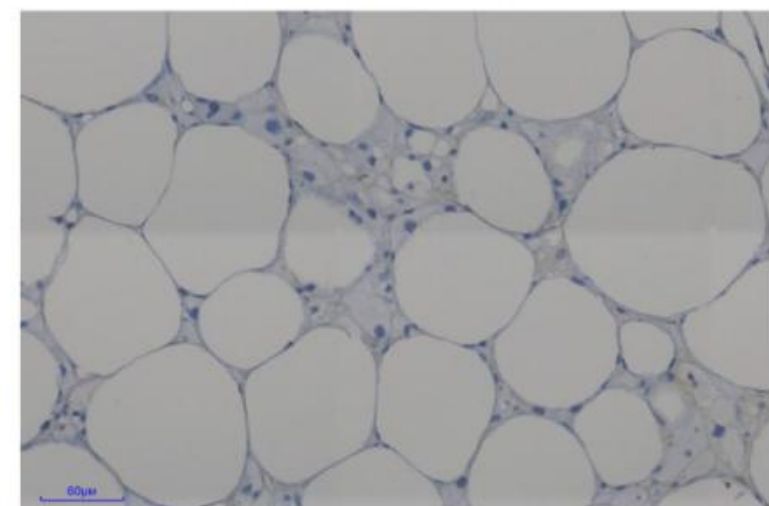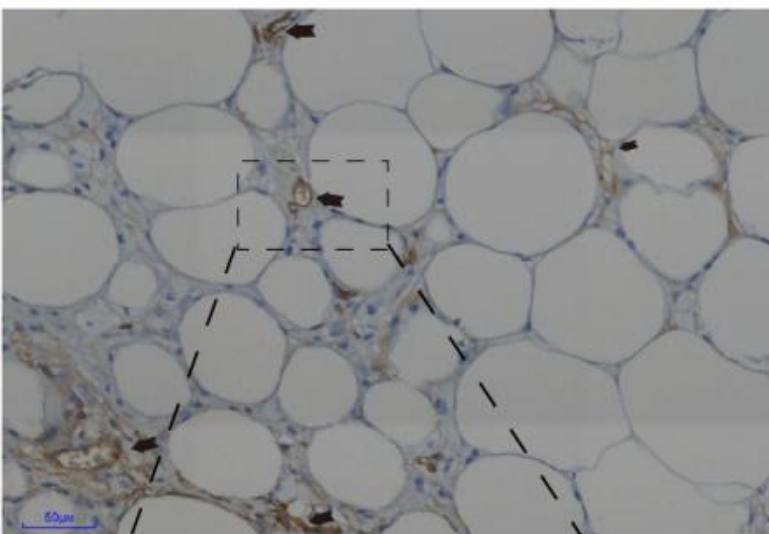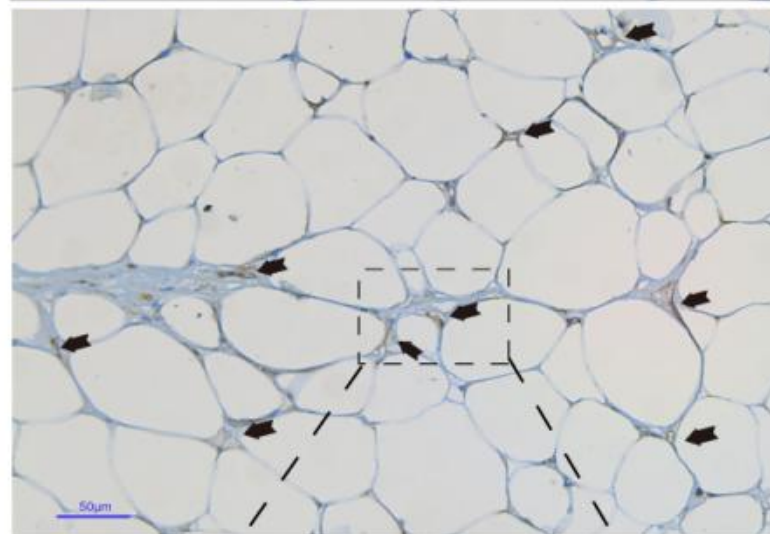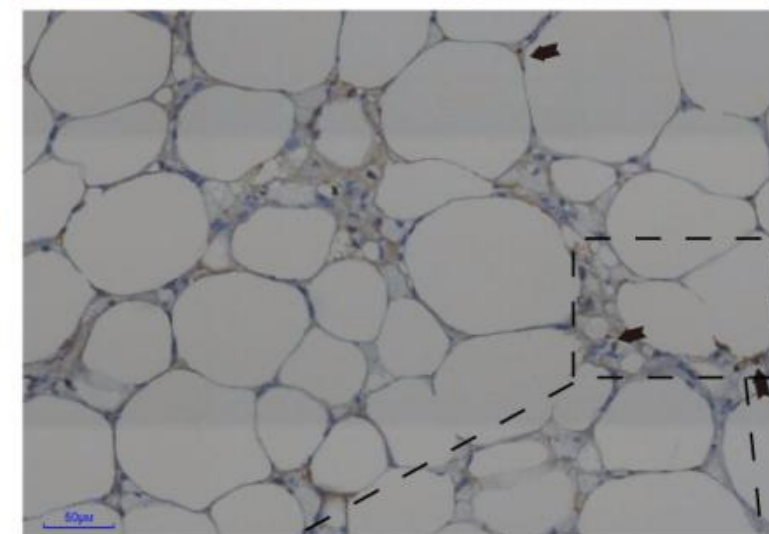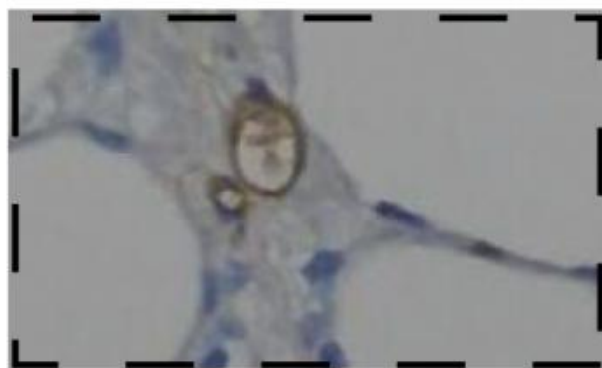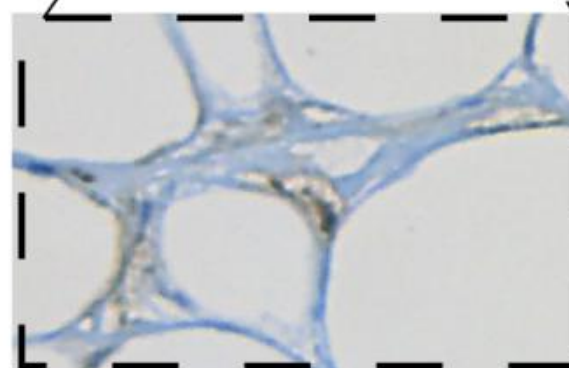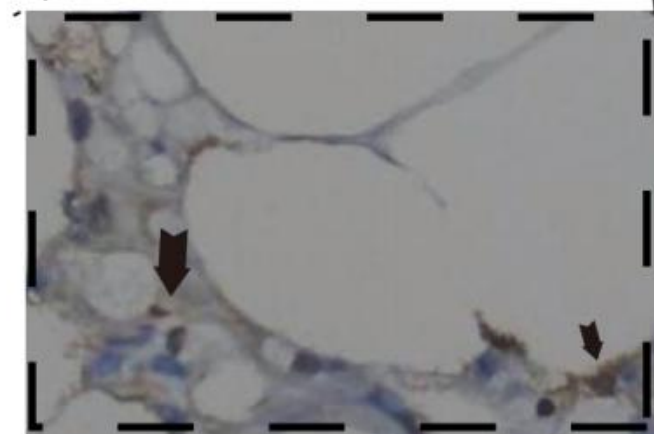

3 Month

PBS

hADMSC-sEVs

CD34

VEGF

Ki-67

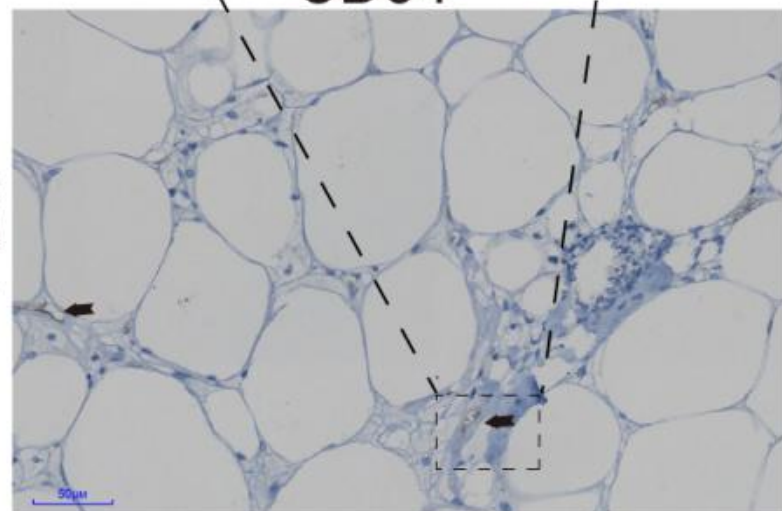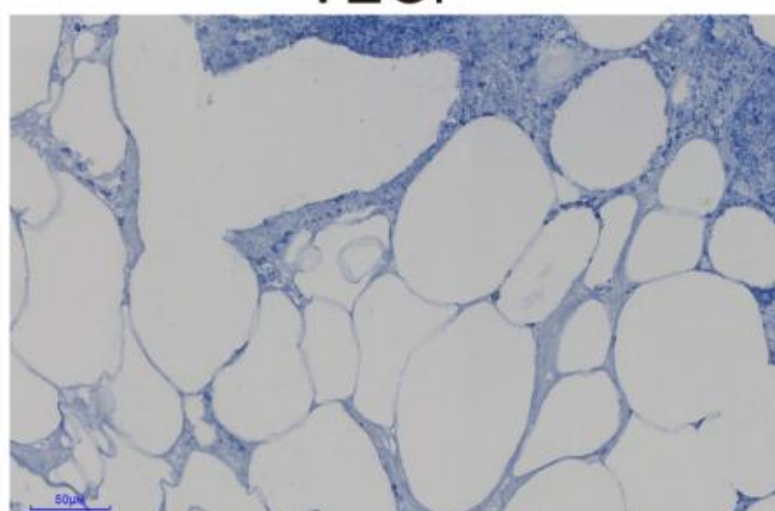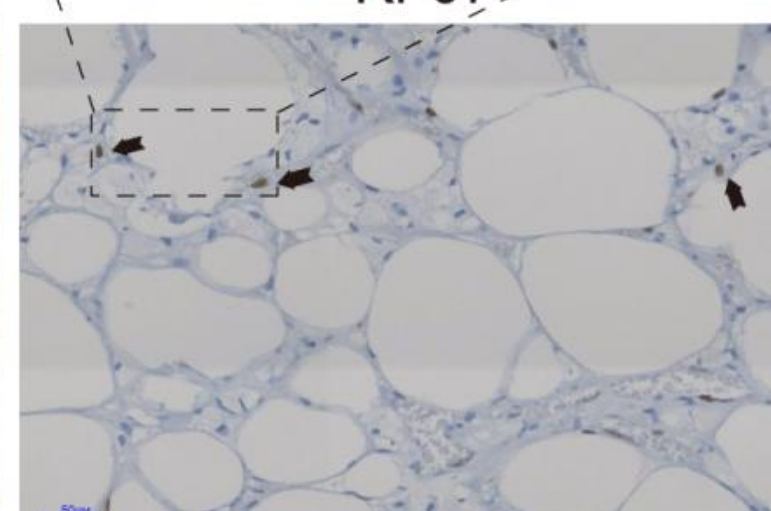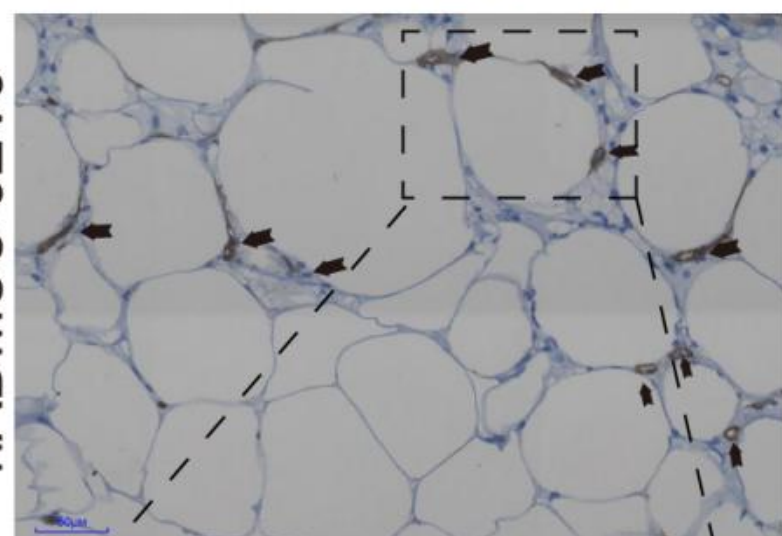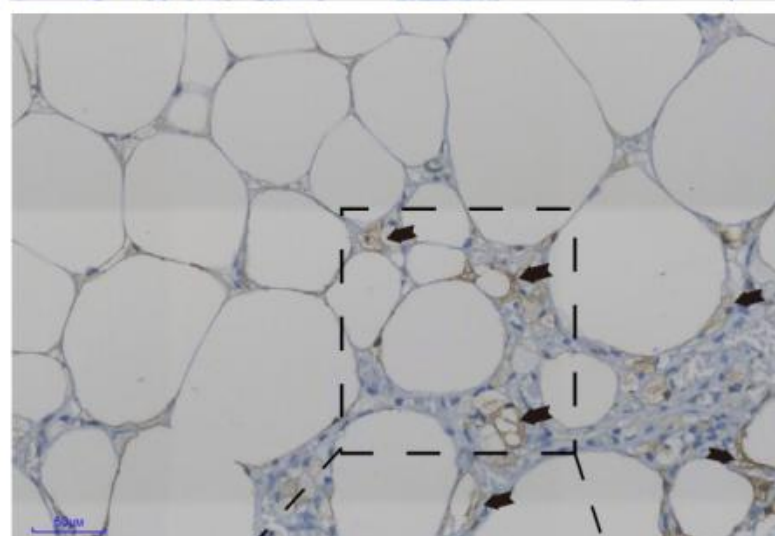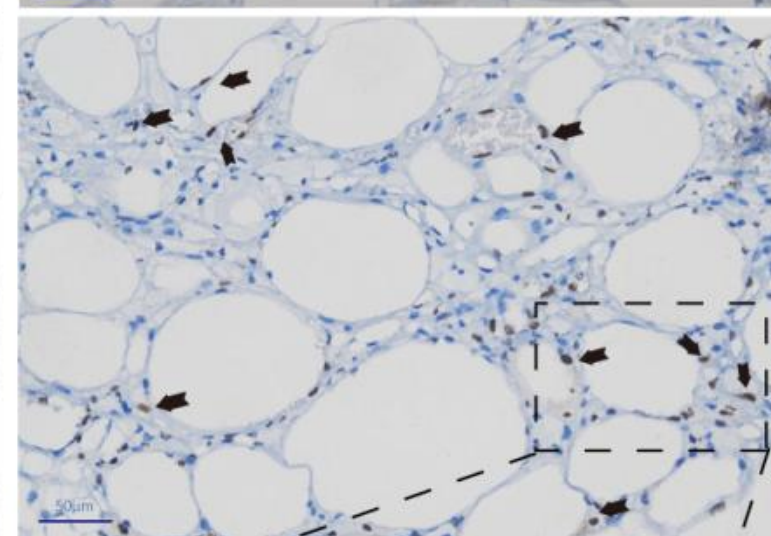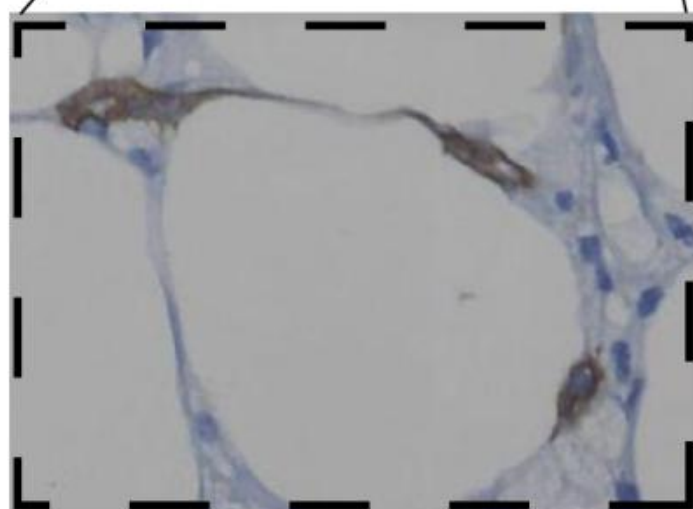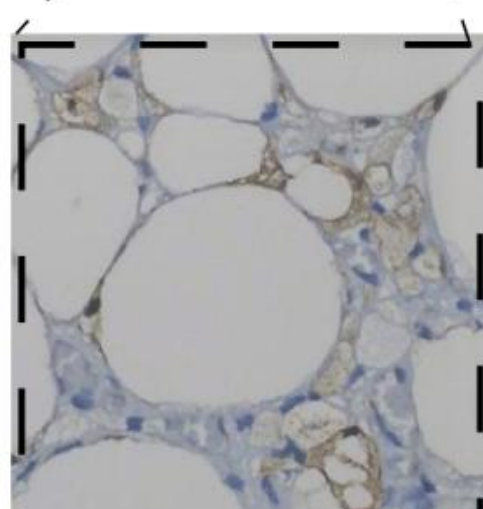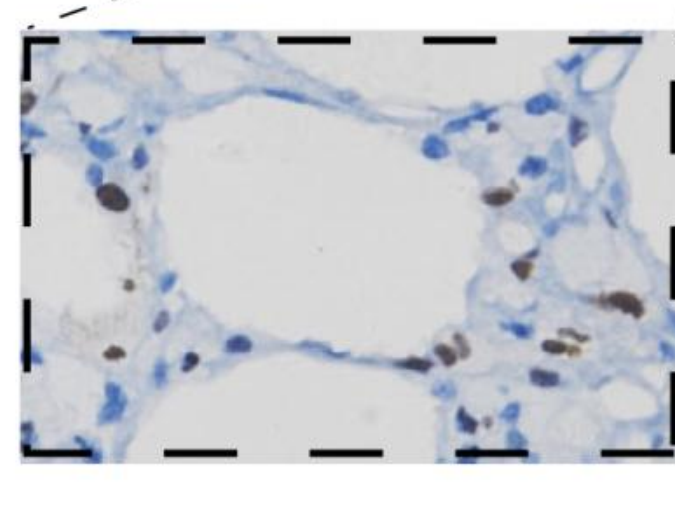

Supplement: Supplementary file 1 — Additional file 1. Supplemntal material (enlarged figures) hADMSC-sEVs promoted neovascularization in the nude mice fat grafting model (rows meaned different staining and lines represents different groups and months). [file 13287_2021_2319_MOESM1_ESM.pdf]
